# Maternal immunity and post-vaccination sero-monitoring in small ruminants for peste des petits ruminants eradication in North Shewa, Ethiopia

**DOI:** 10.1038/s41598-026-41977-3

**Published:** 2026-02-27

**Authors:** Enyiew Alemnew Alamerew, Demeke Sibhatu, Thomas Cherenet, Fasil Aklilu, Derib Aydefruhim, Firdawok Ayele, Asfaw Bisrat, Getachew Hailu, Shenkute Goshme, Erdachew Yitagesu, Yeshitla Wondifra, Meron Moges, Yonas Alemayehu, Melkamu Tadesse, Anmaw Shite Abat

**Affiliations:** 1 Amhara Agricultural Research institute, Debre Birhan Agricultural Research Centre, P.O. Box 112, Debre Birhan, Ethiopia; 2 Departement of Veterinary Pathobiology, University of Gondar, P.O. Box 5689, Godar, Ethiopia; 3 Département of Serology, Animal Health Institute, Sebeta, Ethiopia; 4 Livestock and Fishery Sector Development Project, Ministry of Agriculture, P.O. Box 62347, Addis Ababa, Ethiopia; 5 Disease Prevention and Control, Ministry of Agriculture, P.O. Box 62347, Addis Ababa, Ethiopia

**Keywords:** Antibody, Flock immunity, Risk-based vaccination strategy, Vaccinated small ruminants, Diseases, Immunology, Microbiology

## Abstract

Ethiopia aims to eradicate peste des petits ruminants (PPR) by 2027 through a risk-based vaccination strategy (RBVS); however, outbreaks continue to occur. This cross-sectional study (January–May 2024) evaluated RBVS performance by assessing flock- and maternal-level antibody immunity, providing field-based evidence to inform PPR control and eradication efforts. A total of 594 serum samples were collected using simple random sampling to assess flock (*n* = 508) and maternal (*n* = 86) antibody immunity. All samples were tested for PPRV antibodies using a competitive enzyme-linked immunosorbent assay (c-ELISA). Overall individual animal-level antibody immunity was 65.35% (332/508), while at the flock level only 22.27% of flocks (6/22) had ≥ 80% of animals with detectable antibodies. Multivariable logistic regression showed significant differences in immune status by age group (*p* < 0.05). Adult (284/428, 66.36%) and older (26/33, 78.79%) small ruminants were approximately three and four times more likely to be seropositive than young animals (52.21%), respectively. Significant district-level variation was also observed: animals in Basona-werena (116/146, 79.45%) were three times more likely to be seropositive than those in Menz-mama (88/155, 56.77%). Overall maternal antibody immunity in kids and lambs was 52.3%, with 61.29% (95% CI: 42.19–78.15) in kids and 47.27% (95% CI: 33.65–61.20) in lambs. This study demonstrates insufficient maternal and flock-level antibody immunity against PPRV in vaccinated small ruminants, with levels below the recommended 80% threshold. To improve coverage, vaccination efforts should prioritize young animals, districts with low antibody levels, and areas with poor past performance. Strengthening surveillance, refining vaccination strategies, enhancing sero-monitoring, and ensuring efficient vaccine distribution are essential for effective PPR control and eventual eradication.

**Supplementary Information:**

The online version contains supplementary material available at 10.1038/s41598-026-41977-3.

## Introduction

Peste des petits ruminants (PPR) is an acute transboundary viral disease that affects both domestic and wild small ruminants^1^. The causative agent, *Morbillivirus caprinae* (formerly PPR virus (PPRV)), is a negative-sense single-stranded RNA virus belonging to the genus *Morbillivirus* within the family *Paramyxoviridae*^2^. Clinically, PPR is characterized by pyrexia, oral erosions, ocular and nasal discharge, enteritis, diarrhea, and bronchopneumonia^3^.

PPR was first reported in Côte d’Ivoire in 1942 and has since spread extensively across sub-Saharan Africa, the Arabian Peninsula, the Middle East, Asia, and parts of Europe^4–6^. In Ethiopia, the disease was initially suspected in a goat flock in the Afar region in 1977 and has since become one of the country’s most economically significant livestock diseases^7–14^, often causing high morbidity and mortality rates^15^. In fully susceptible populations, infection can involve up to 100% of animals, with mortality ranging from 10% to 90%^16^. Globally, approximately 30 million animals are affected by PPR across 70 countries^17^, resulting in estimated annual economic losses of up to USD 2.1 billion^18^. Specifically, in Ethiopia, PPR is reported to cause an average loss of USD 375 per flock, with over 143 small ruminants per flock affected (equivalent to more than USD 2 loss per animal)^19^. These impacts have profound implications for food security and the livelihoods of rural communities, particularly in sub-Saharan Africa, where small-ruminant farming constitutes a crucial component of local economies^20^.

To mitigate these effects, a global initiative for progressive PPR control was launched in 2015, aiming for worldwide eradication by 2030^3^. In line with this objective, the Food and Agriculture Organization (FAO) and Ethiopia’s Ministry of Agriculture (MoA) committed to eradicating PPR in Ethiopia by 2027^21^. Central to these efforts is the use of effective PPR vaccines^22,23^. While achieving 100% vaccination coverage is ideal (23), effective herd protection can be realized when at least 50% of vaccinated units achieve a minimum of 80% flock immunity^17^. In Ethiopia, RBVS have been implemented since September 2018; nevertheless, PPR outbreaks have persisted, with 554 reported nationwide between 2018 and 2022^13,24^. The ongoing circulation of PPRV in Ethiopia and other regions worldwide^4,5,7–14,25,26^ highlights the challenges in controlling the disease and underscores the need to strengthen surveillance, optimize vaccination strategies, improve sero-monitoring, and ensure efficient vaccine distribution^17,27^.

Despite these efforts, specific data on risk-based vaccination campaigns in Ethiopia remain scarce, limiting the planning and implementation of effective vaccination programs^13,28^. Moreover, comprehensive studies examining the socioeconomic impact of PPR and the effectiveness of vaccination interventions in endemic areas are limited^3^. Epidemiological investigations, including post-vaccination sero-monitoring to evaluate vaccine efficacy and immune status, are critical for guiding PPR eradication efforts^24,29^. To the authors’ knowledge, no studies have assessed the progress of PPR RBVS in the North Shewa Zone of Ethiopia. Therefore, this study aims to fill this gap by evaluating the current status vaccination campaigns. Specifically, it assesses maternal and flock antibody immunity in vaccinated small ruminants across selected districts of North Shewa Zone and identifies factors associated with individual animal level immunity in these populations.

## Materials and methods

### Description of the study area

**Fig. 1:**
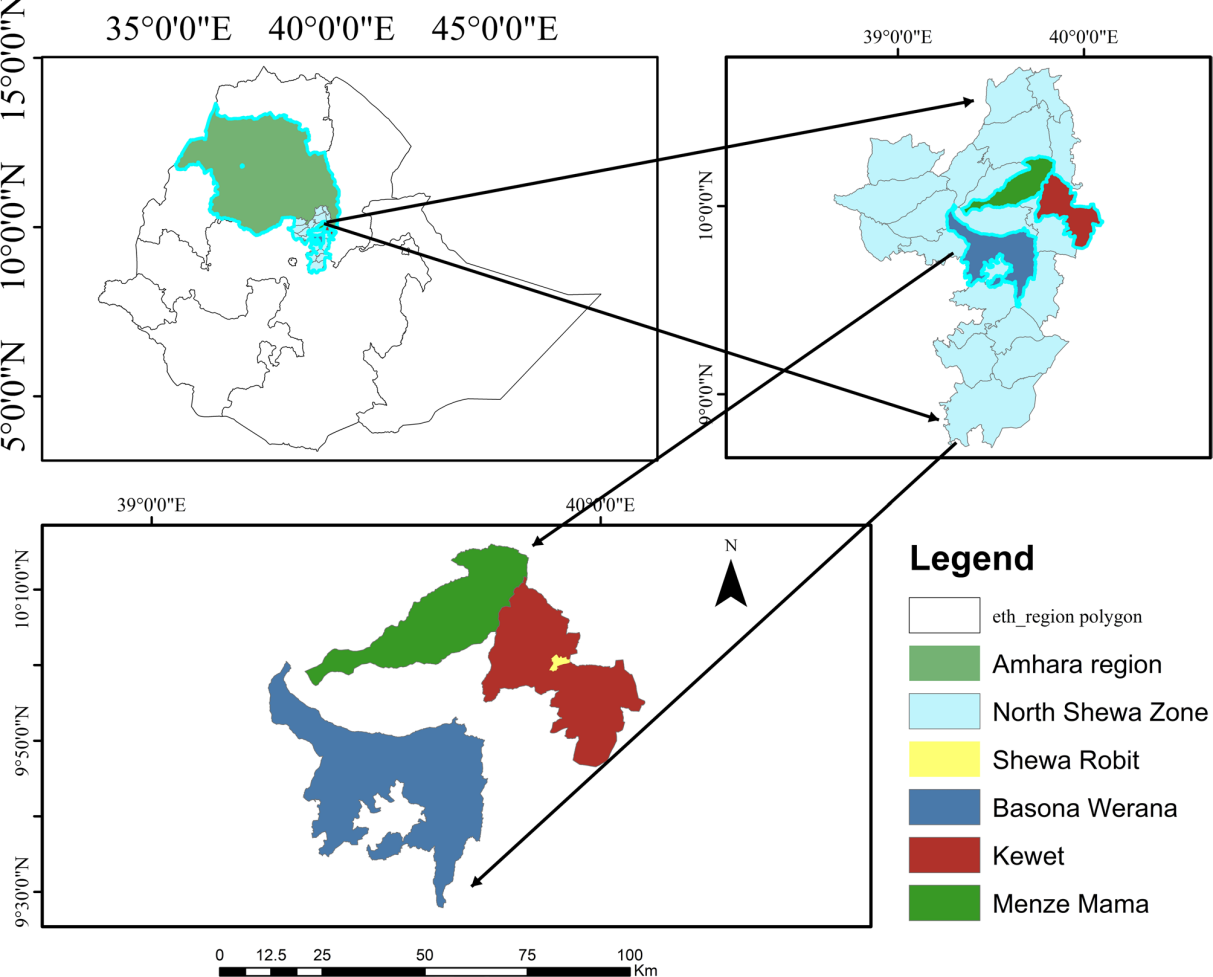
Map of the study areas.

The current study was conducted in four selected districts of the North Shewa Zone, Amhara Region, Ethiopia: Basona-werana, Menz-mama, Kewet, and Shewa-robit. Kewet and Shewa-robit are located 200 km and 240 km from Addis Ababa, respectively, and are situated at 10°21’0” N and 39°55’60” E, with elevations ranging from 1280 to 1468 m above sea level (Fig. 1). These areas experience a bimodal rainfall pattern, with a long-wet season from June to September, a short rainy season from February to May, and a dry season from October to January. The average annual rainfall ranges between 1085 and 1199 mm, and the temperature varies from 25.4 °C to 27.0 °C, with June being the hottest month and December being the coolest^30^. The Basona-werana and Menz-mama districts, located 110–130 km and 270–301 km north of Addis Ababa, respectively, lie at elevations above 2770 and 3132 m. Basona-werana receives 950–1200 mm of rainfall annually, with temperatures ranging from 1.5 to 23.3 °C, whereas Menz-mama receives approximately 1149 mm, with an average temperature of 12.2 °C. Both districts experience bimodal rainfall, with short rainy seasons occurring between December and March and long rainy seasons spanning June to November, varying slightly in timing^31^.

### Study population

The study population comprised sheep and goats managed under traditional smallholder systems, where PPR vaccination is typically administered to animals aged three months and older. Between March and August 2023, the animals received the attenuated homologous Nigerian 75/1 vaccine (National Veterinary Institute, Debre-Zeit, Ethiopia). Inclusion in the vaccinated group required documented proof of vaccination, which was verified through official records, district-level veterinary registers, confirmation from animal owners collected via structured and semi-structured questionnaires, and a retrospective dataset obtained from the North Shewa Zone Animal Health Office through the Disease Outbreaks and Vaccination Activity Reports (DOVAR) system, ensuring accurate and comprehensive vaccination documentation (Annex 1). To assess maternal immunity, animals aged 0–3 months (0–90 days), presumed to possess maternal antibodies, were included. In contrast, animals aged 3–6 months (91–180 days) were excluded from sero-monitoring, as they may have initially fallen within the 0–3 month category during vaccination but could have experienced a decline in maternal antibodies by the time of testing^17^. Small ruminants introduced after the last vaccination date, whether through gifts, breeding, sharing, or purchase, were excluded from the study because their vaccination history was unknown. Lambs and kids born to mothers with an unknown vaccination history were also excluded from the study.

### Study design and sampling procedure

A cross-sectional study was carried out in four districts from January to May 2024 to collect blood samples from small ruminants. A multistage cluster sampling technique was employed for the study. In the first stage, the North Shewa zone was purposively selected because it had implemented the PPR-RBVS program^21^. In the second stage, the 24 districts within the zone were categorized on the basis of vaccination status: those with and without vaccination programs. Only districts with vaccination programs were included in the third stage. In the third stage, four districts, Kewet, Shewa-robit, Basona-werena, and Menz-mama, were randomly selected. In the fourth stage, two peasant associations (PAs) were randomly selected from each district. In the fifth stage, villages were randomly chosen within each PA. In the final stage, the animals were selected using a simple random sampling method.

### Sample size estimation

The sample size for the study was determined using the following formula^31^:

1 $$n=mc=\frac{\mathrm{P}\left(100-P\right)D}{{SE}^{2}}$$

where n is the sample size, P is the prevalence as a percentage, D is the design effect, SE is the precision, C is the number of clusters (flocks) sampled, and m is the average number of animals sampled per flock (cluster). The design effect was determined using the following formula^32^:

2 $${\mathrm{D}}={\mathrm{1}}+\rho \left( {{\mathrm{m}} - {\mathrm{1}}} \right)$$

where ρ is the intracluster correlation coefficient. A flock of sheep or goats in a village was considered a cluster. The estimate for ρ in most infectious diseases rarely exceeds 0.20^33^. Thus, assuming a value of ρ = 0.20, the average number of animals to be sampled per cluster was estimated to be 27.

3 $${\mathrm{m}}=\sqrt {{\mathrm{C}}1/{\mathrm{C}}2 \times (1 - \rho )/\rho }$$

where C1 is the cost of sampling a cluster and C2 is the cost of sampling a sheep or a goat within a cluster. C1 was estimated to be 15 times greater than C2 by considering the distance between the clusters. The design effect (D) of a cluster-sample survey with a sample of 27 animals per cluster is 6.2 (Formula 2). On the basis of an expected immune response of 61%^34^ and a desired precision of 5%, sampling 27 animals per cluster required 22 clusters, resulting in a total sample size of approximately 594 animals.

A total of 22 villages were selected proportionally on the basis of small ruminant population density in each district: 4 from Shewa-robit, 5 from Kewet, 6 from Basona-werana, and 7 from Menz-mama. In each village, all the small ruminants shared common grazing and watering points, forming a single cluster (a single flock). Lists of PAs, along with records of vaccinated and unvaccinated flocks, were obtained from zonal and district agricultural offices and verified with PA development agents. Vaccination status was confirmed using these official records. The most recent PPR vaccination campaigns were conducted in August 2023 for Kewet and Shewa-robit, July 2023 for Basona-werana, and April 2023 for Menz-mama. Sample sizes were proportionally allocated on the basis of the small ruminant population in each district, following RBVC guidelines.

### Sample collection

Approximately 5 mL of blood was collected from selected vaccinated small ruminants and their offspring by direct jugular venipuncture using sterile vacutainer systems. Animals were manually restrained during sampling. The blood samples were allowed to clot for 30–60 min at room temperature before transport to the laboratory, as sampling sites were distant from the laboratory and lacked an immediate power supply. Serum was then separated by centrifugation at 3,000 rpm. The separated serum was transferred into sterile cryovials, properly labeled, and stored at − 20 °C until testing. During sampling, relevant information for each animal, including origin (district, PA, and village), age, sex, species, and body condition score, was recorded (Annex 1). Body condition scores of sheep and goats were categorized as poor, medium, or good^35^. Age was determined based on dentition and classified as young (6–12 months), adult (1–5 years), and old (> 5 years)^36,37^.

### Serological examination

The samples were tested for PPR virus antibodies at the AHI laboratory in Sebeta, Ethiopia, using the ID Screen^®^ PPR Competition ELISA Kit (IDvet Screen^®^ PPR Competition Kit, 310 Rue Louis Pasteur, 34790 Grabels, France). This method detects antibodies against the PPR virus nucleoprotein using a validated ELISA kit. Optical density (OD) was measured at 450 nm with an ELISA plate reader, and sample positivity or negativity was determined by calculating the sample-to-negative (S/N) ratio according to the manufacturer’s instructions as follows:

4 $$\frac{\mathrm{S}}{\mathrm{N}}=\frac{\mathrm{O}\mathrm{D}\mathrm{S}}{\mathrm{O}\mathrm{D}\mathrm{N}\mathrm{C}}\times 100$$

where ODS represents the sample optical density and ODNC represents the negative control optical density. Samples with S/*N* ≤ 50% were considered positive, those with 50% < S/*N* ≤ 60% were classified as doubtful, and those with S/*N* > 60% were considered negative. About eight samples with doubtful results were retested, and only clearly positive or negative samples were included in the statistical analyses.

### Data management and statistical analysis

Data from field sampling and laboratory analyses were entered into Microsoft Excel for management, visualization, and computation of antibody immunity at the individual, flock, and maternal levels against PPR. Individual animal-level antibody immunity was calculated as the proportion of seropositive animals among the total tested. Flock-level immunity was calculated as the proportion of seropositive animals within each village, with each village considered a single flock since animals shared common grazing and watering points. Flock-level protective immunity was defined as villages achieving ≥ 80% seroprevalence, based on the herd immunity theory.

5 $$H=1-\frac{1}{{R}_{0}}$$

where H is the herd immunity threshold and R_0_ is the basic reproduction number. Published R_0_ estimates for PPRV in small ruminants range from 3 to 7, corresponding to flock immunity thresholds of 67–86%. For this study, R_0_ = 5 was used, giving a threshold of approximately 80%, consistent with FAO–WOAH Global PPR Eradication Programme guidance. Overall flock-level immunity was calculated as the proportion of villages meeting this threshold relative to the total number of villages surveyed^38–41^.

Statistical analyses were conducted using STATA 17. Univariable logistic regression was used to identify associations between seropositivity and potential risk factors (*p* < 0.25), which were then further examined using forward and backward stepwise multivariable logistic regression. Cramér’s V, a measure of association, was applied to exclude highly correlated variables (*r* ≥ 0.7) from the multivariable models. Two-way interactions and confounding effects, defined as a greater than 30% change in beta coefficients, were also assessed. Statistical significance was set at *p* < 0.05 with 95% confidence intervals.

## ResultS

### Flock immunity

The overall individual animal-level antibody immunity across the study districts was 65.35% (332/508 serum samples), which is below the threshold (80%) required to reduce infection transmission sufficiently to eliminate the virus. Among districts, the highest immunity was observed in Basona-werena (79.45%), while the lowest was in Menz-mama (56.77%) (Table 2). At the PA level, Abamote showed the highest immunity at 82.7%, exceeding the threshold, whereas Keyafer had the lowest at 50.75% (Table 1). At the flock level, only 6 of the 22 flocks (22.27%) had ≥ 80% of animals with detectable antibodies. The highest flock-level antibody immunity was observed in Arkisekelo (92.74%, 95% CI: 74–99%), while the lowest was in Keyafer (22.73%, 95% CI 8–45%) villages (Annex 2 Table S1).

**Table 1:** Individual level antibody immunity against PPRV in vaccinated Small ruminant across study areas.

Districts	Peasant associations	No. sampled	No. positive	Prevalence (%)	Confidence interval (95%)	
					Lower	Upper
Basona-werena	Abamote	75	62	82.67	72.19	90.44
	Kormargefiya	71	54	76.06	64.46	85.39
Kewet	Tere	71	52	73.24	61.41	83.06
	Yelen	38	20	52.63	35.82	69.02
Shewa-robit	Wustimbay	47	23	48.94	34.08	63.94
	Wanza	51	33	64.71	50.07	77.57
Menz-mama	Zeram	88	54	61.36	50.38	71.56
	Keyafer	67	34	50.75	38.24	63.18
Total		508	332	65.35	61.04	69.49

Univariable logistic regression analysis revealed significant differences (*p* < 0.05) in immune status among the age groups: adult (66.36%) and old (78.29%) small ruminants had two- and fourfold greater odds of seropositivity, respectively, than younger animals did (52.21%). Additionally, small ruminants in the Basona-werena district (79.45%) had three times greater odds of seropositivity than those in Menz-mama (56.77%) (Table 2).

**Table 2:** Univariable logistic regression model for risk factors influencing the immune response to PPRV vaccination in vaccinated small ruminants.

Risk factors	Risk factors category	No. sampled	No. positive	Flock immunity (%)	*p* value	Odds ratio	CI (95%)	
							Lower	Upper
Districts	Menz-mama*	155	88	56.77	**–**	**–**	**–**	**–**
	Shewa-robit	98	56	57.14	0.954	1.02	0.61	1.69
	Kewet	109	72	66.06	0.129	1.48	0.89	2.46
	Basona-werena	146	116	79.45	0.000	2.94	1.76	4.91
Species	Goat*	194	118	60.82	**–**	**–**	**–**	**–**
	Sheep	314	214	68.15	0.092	1.38	0.95	2.01
Age	Young*	47	22	46.81	**–**	**–**	**–**	**–**
	Adult	428	284	66.36	0.009	2.24	1.22	4.11
	Old	33	26	78.29	0.005	4.22	1.53	11.62
Sex	Female*	478	312	65.27	**–**	**–**	**–**	**–**
	Male	30	20	66.67	0.876	1.06	0.49	2.33
Body condition scores	Good*	270	173	64.07	**–**	**–**	**–**	**–**
	Moderate	190	128	67.37	0.465	1.16	0.78	1.71
	Poor	48	31	64.58	0.946	1.02	0.54	1.94
Agro-ecology	Lowland*	207	128	61.84	**–**	**–**	**–**	**–**
	Highland	301	204	67.77	0.167	1.3	0.9	1.88
Total		508	332	65.35				

*Reference, OR: odds ratio; CI: confidence interval.

For the multivariable model (*p* < 0.25), study districts, species, age, and agroecological factors related to PPRV were selected (Table 2). Cramér’s V statistic indicated no correlation between pairs of independent variables, and confounding factors were assessed during model building, but no confounding variables were found. All two-way interactions were tested, but none were found to be significant. Ultimately, the final multivariable logistic regression analysis revealed significant differences (*p* < 0.05) in antibody immune status among the age groups: adult (66.36%) and old (78.29%) small ruminants were approximately three and four times more likely to be seropositive than young individuals were (52.21%), respectively. Additionally, significant variation was observed among the study districts, with small ruminants in the Basona-werena district (79.45%) being three times more likely to be seropositive than those in the Menz-mama district (56.77%) (Table 3).

**Table 3:** Multivariable logistic regression model for potential risk factors influencing the immune response to PPRV vaccination.

Risk factors	Category of risk factors	*p* value	Odds ratio	95% Confidence Interval	
				Lower	Upper
Districts	Menz-mama*	**–**	**–**	**–**	**–**
	Shewa-robit	0.549	0.89	0.53	1.52
	Kewet	0.184	1.35	0.8	2.28
	Basona-werena	0.000	2.98	1.76	5.02
Age	Young*	**–**	**–**	**–**	**–**
	Adult	0.002	2.78	1.45	5.34
	Old	0.010	3.93	1.39	11.11

*Reference, OR: odds ratio.

### Maternal immunity

A total of 86 animals aged three months or younger and born to vaccinated dams were assessed to evaluate maternal antibody immunity. The overall seropositivity among these young animals was 52.33%. The highest seropositivity rate was observed in the ≤ 30-day age group (67.65%, 95% CI 49.47–82.61), whereas the lowest was recorded in the 61–90-day age group (35.71%, 95% CI 18.64–55.95), indicating a decline in maternal antibody immunity as age increased. Goat kids were more seropositive (61.29%, 95% CI 42.19–78.15) than lambs were (47.27%, 95% CI 33.65–61.20). In terms of sex, male offspring had slightly greater seropositivity (53.85%, 95% CI 33.37–73.41) than did female offspring (51.67%, 95% CI 38.40–64.77) (Table 4).

**Table 4:** Maternal immunity in lambs and kids born to PPRV-vaccinated small ruminants.

Variables	Category	No. of sampled	No. positives	Proportions (%)	Confidence interval (95%)	
					Lower	Upper
Age	61–90 days	28	10	35.71	18.64	55.95
	31-60days	24	12	50	29.12	70.88
	<= 30days	34	23	67.65	49.47	82.61
Species	Sheep	55	26	47.27	33.65	61.2
	Goat	31	19	61.29	42.19	78.15
Sex	Female	60	31	51.67	38.4	64.77
	Male	26	14	53.85	33.37	73.41
Total		86	45	52.33	41.27	63.21

## Discussion

### Flock immunity

Post-vaccination sero-monitoring, using outbreak reports, participatory disease searches, and sero-epidemiological surveys, is essential for evaluating vaccination effectiveness and strengthening PPR surveillance to enable early outbreak detection, monitor vaccine performance, and track progress toward eradication^17,27^. In North Shewa Zone, Amhara Region, Ethiopia, nearly three million small ruminants were vaccinated through RBVS between 2018 and 2024. Despite these extensive efforts, persistent outbreaks were reported in the zone during this period^24^. Achieving high population immunity through vaccination is critical for reducing outbreak risk, limiting transmission, and ultimately eradicating PPRV^17,41^. Accordingly, WOAH–FAO recommends attaining at least 80% flock immunity to protect susceptible populations^17^, although experiences such as Morocco’s eradication program suggest that herd immunity above 70% may still substantially restrict virus spread^42^.

In the study zone, confirmation of PPR outbreaks is followed by targeted control measures, including isolation of at-risk populations and emergency vaccination with live attenuated PPR vaccine, prioritizing high-risk areas^6,43^. These interventions demonstrate strong institutional commitment to disease control and form a core component of the risk-based vaccination campaign implemented across the zone^6,43,44^. Within this framework, the present study provides important evidence on post-vaccination antibody immunity and offers insights into the performance of these control efforts. The study documented an overall individual-level antibody immunity of 65.35% (95% CI 61–70%), below the recommended 80% flock immunity threshold generally required to limit PPRV transmission. At the flock level, only 6 of the 22 flocks (22.27%) had ≥ 80% of animals with detectable antibodies. Although these levels indicate meaningful progress toward building population immunity, interpretation should consider that, in the absence of a DIVA-compatible vaccine, seropositivity cannot distinguish vaccine-induced immunity from immunity acquired through natural infection. Given the frequent outbreaks reported in the area^43^, the observed antibody levels likely reflect a combination of both sources. Additionally, exclusion of animals aged 3–6 months may have slightly overestimated flock antibody immunity, which should be considered when interpreting these results.

This finding aligns with previous studies in Ethiopia, such as a 64.5% individual antibody immunity level in vaccinated small ruminants from the eastern Amhara region bordering Afar^45^, a 61% seroconversion rate in the Awash Fentale district of the Afar Region^34^, and a 68.8% seroconversion rate in the Borena zone^13^. These studies suggest a consistent range of herd immunity levels across different regions of the country. However, these immunity levels fall short of the recommended thresholds^17,42^. In contrast, higher seroconversion rates have been reported in other areas, such as 93.9% in the Metema district of northwest Ethiopia^46^, 86.3% in North Shewa^47^, and 76.66% in the West Gojjam Zone, Ethiopia^48^. The lower antibody immunity level observed in this study and some previous studies may be attributed to logistical challenges in vaccination efforts, such as inadequate cold chain facilities, improper vaccine handling, suboptimal distribution, and limited awareness during vaccination campaigns^46,48–50^. Additionally, the quality of the vaccine itself is a key factor in determining immune outcomes. Singh and Bandyopadhyay^51^ emphasized the importance of using high-quality vaccines with proven efficacy to ensure long-lasting immunity, highlighting the need for stringent quality control measures in vaccination campaigns.

The current study highlights the significant variability in PPRV antibody immunity levels across PAs and districts in North Shewa. For instance, Abamote PA reported a high antibody immunity level of 82.7%, whereas Keyafer reported a much lower rate of 50.8%. The low antibody immunity in Keyafer underscores the difficulties faced by remote or underserved areas in achieving adequate vaccination. These disparities may result from several factors, including inconsistencies in vaccine handling and cold chain maintenance, differences in vaccinator training and technique, and variations in the timing and reach of vaccination campaigns. Flock management practices and the movement of animals can further influence immune outcomes. Additionally, in endemic areas, natural exposure to PPRV may enhance immunity independently of vaccination^46,48–50^. Although this study did not directly assess these factors, their potential impact is acknowledged, and further investigation is recommended to address the causes of uneven immunity. Similar variability has been reported elsewhere. In Ethiopia, flock-level immunity ranges from 65.5% to 96.1%^52^. In Kenya, immunity levels among small ruminants vary from 50% to 80% and are largely influenced by the extent and consistency of vaccination efforts^53^. A comparable pattern is observed in Somalia, where the success of vaccination campaigns has been inconsistent^54^. These findings collectively highlight the broader challenges of achieving comprehensive PPR control across the region, particularly in the face of logistical, economic, and social barriers. Addressing these challenges is essential for ensuring effective and sustained disease control in Ethiopia.

In this study, age was found to have a significant effect on antibody immunity levels. Compared with their younger counterparts, adult small ruminants (66.4%) were nearly twice as likely, and older animals (78.3%) were nearly four times as likely to be seropositive. This suggests that older animals may benefit from repeated vaccinations or natural exposure to infections, a pattern also observed in previous studies^55,56^. The study also revealed significant variation in seropositivity rates across districts. Small ruminants in Basona-werena (79.5%) were nearly three times more likely to be seropositive than those in Menz-mama district (56.8%). This geographical disparity may be influenced by local factors such as animal management practices and environmental conditions. These findings align with previous research indicating that socioeconomic conditions, local disease prevalence, and governance infrastructure significantly impact vaccination uptake and effectiveness^3^. These findings highlight the importance of considering age- and district-specific factors when designing targeted vaccination programs. These findings are consistent with the recommendations of previous studies, which emphasize the need to align vaccination efforts with local epidemiological data to enhance disease control. Comprehensive vaccination strategies, supported by ongoing monitoring of immune responses, are essential for the long-term success of PPRV eradication initiatives^41,57^.

### Maternal immunity

Maternal immunity plays a vital role in protecting young ruminants from PPRV, supporting efforts toward its control and eradication^56^. Evaluating maternal antibodies and vaccination programs is critical for identifying strategies to strengthen immunity in young ruminants, improve flock health, and reduce the prevalence of PPRV^58^. In this study, the overall individual animal level maternal antibody immunity among lambs and kids born to PPRV-vaccinated dams was 52.3%. Although a global standard for maternal antibody protection against PPRV is lacking, a widely accepted benchmark, which is based on WOAH guidelines, field studies, and best practices in PPR control, suggests that at least 80% of offspring born to vaccinated dams should have detectable maternal antibodies during the first 2–3 months of life. This finding is consistent with previous reports that documented maternal antibody immunity levels ranging from 40% to 60% in lambs and kids^53^. Similarly, maternal immunity often fails to reach optimal levels, emphasizing the need for improved vaccination strategies to enhance protection for young ruminants^3^. Several factors may contribute to this suboptimal immunity, including poor nutrition, weak immune responses in dams, substandard vaccine quality, and environmental stressors^59^. Factors such as poor nutrition and disease exposure hinder antibody production and transfer^60^. Additionally, in regions with limited veterinary infrastructure, achieving the necessary immunity levels for effective flock protection remains a significant challenge. To improve maternal immunity, increasing vaccination coverage, enhancing vaccine quality, and implementing targeted vaccination strategies for young ruminants could be valuable approaches^17^.

A key finding of this study is the decline in maternal antibody immunity with increasing offspring age, with the highest antibody immunity observed in the ≤ 30 days group (67.7%) and the lowest in the 61–90 days group (35.7%). A similar trend was reported, with a decrease in maternal antibody levels between 42 and 100 days in lambs and kids^60–65^. Additionally, maternal immunity in kids decreases below protective levels after four months^66^. Furthermore, antibody titers from live attenuated PPR vaccines significantly decline after six months^66,67^. These findings suggest that maternal immunity becomes insufficient beyond 2–6 months of age, even with successful maternal vaccination. As PPR vaccination provides early protection, immunity decreases over time, increasing the vulnerability of offspring to infection^68^. Variations in the immune response may arise from factors such as colostrum antibody levels, absorption efficiency, vaccine strain, and species and breed differences^69,70^. These results underscore the need for timely vaccination strategies to maintain protection during early life stages and highlight the importance of understanding maternal immunity dynamics to design more effective vaccination schedules for small ruminants.

Although higher maternal antibody levels were observed in goat kids (61.3%) than in lambs (47.3%), the difference was not statistically significant because of overlapping confidence intervals and limited subgroup sizes. Therefore, these findings should be interpreted with caution and considered preliminary. This trend aligns with a previous study, which reported that at 105 days of age, 80% of kids retained protective immunity, whereas only 40% of lambs did^61^. This study also revealed a decrease in maternal antibodies, with immunity waning by 120 days in lambs and 150 days in kids. Additionally, goats have been shown to exhibit higher levels of PPRV-specific antibodies than sheep do^10,71,72^, and a higher percentage of goats seroconverted following exposure or vaccination than sheep did (24). Overall, the confidence intervals for age group, species, and sex overlapped considerably, indicating that the observed differences were not statistically significant. Furthermore, the small sample size limited the ability to draw strong conclusions regarding differences among the study groups.

In general, post-vaccination an individual animal and flock level and maternal antibody immunity in North Shewa Zone remain below recommended thresholds, with significant variation by age and location. These findings underscore the need for targeted, risk-based vaccination strategy, ongoing sero-monitoring, and consideration of maternal antibody dynamics to strengthen flock immunity and advance PPR control and eradication efforts.

### Limitations of the study

This study was conducted as a cross-sectional survey due to logistical and resource constraints, which prevented comparisons with non-vaccinated animals, as regional vaccination campaigns targeted all eligible small ruminants. Post-vaccination antibody immunity was assessed through circulating antibodies, which reflect immunological response but do not confirm clinical protection. In the absence of a DIVA-compatible vaccine, seropositivity cannot reliably distinguish between vaccine-induced and infection-derived immunity; with ongoing outbreaks, the observed antibodies likely represent a combination of both. Animals aged 3–6 months were excluded because of their transitional immune status, potentially introducing bias and slightly overestimating overall flock immunity. Maternal immunity was evaluated in a relatively small sample, further subdivided by species and sex, limiting statistical power to detect significant differences. These factors warrant cautious interpretation of the findings, and future studies with larger, more representative cohorts are recommended to better characterize post-vaccination and maternal antibody dynamics. Despite these limitations, the current study indicates meaningful progress toward building population immunity.

## Conclusion and recommendations

This study revealed that overall individual animal- and flock-level antibody immunity against PPRV in vaccinated small ruminants in North Shewa Zone remains below the threshold required to reduce infection transmission sufficiently to eliminate the virus. Substantial variation in immunity was observed across districts and age groups. Maternal antibody immunity was also low and declined markedly with increasing age. These findings indicate that current risk-based vaccination efforts have not yet achieved adequate population antibody immunity and that immunity gaps persist, particularly among young animals. Despite these challenges, the results demonstrate meaningful progress and strong regional commitment to PPR eradication. However, existing efforts remain insufficient to achieve uniform immunity, underscoring the need for targeted improvements. Based on this evidence, the following recommendations are proposed to strengthen Ethiopia’s PPR eradication strategy, enhance disease control measures, and support the global eradication campaign:

- Targeted vaccination of young animals should be implemented through systematic follow-up vaccination of lambs and kids when maternal antibodies wane. Young stock should be explicitly included in both routine and supplementary vaccination campaigns to reduce immunity gaps.
- District-level prioritization guided by sero-monitoring should be adopted, with districts exhibiting lower immunity levels prioritized for intensified vaccination, supervision, and resource allocation. District-level serological data should be routinely used to guide vaccination planning and coverage targets.
- Routine post-vaccination immunity assessment should be integrated into vaccination programs through institutionalized periodic sero-monitoring to evaluate vaccine performance and population immunity. Feedback mechanisms should be established to ensure that sero-monitoring results directly inform subsequent vaccination strategies.
- Surveillance and vaccination data systems should be strengthened by improving integration between vaccination records and disease surveillance platforms to enable timely identification of immunity gaps. These integrated data should support evidence-based decision-making at district and zonal levels.
- Further studies on vaccine quality, cold chain integrity, vaccination coverage, and program implementation are recommended to identify the underlying causes of uneven immunity across regions and to guide refinement of vaccination strategies.

## Supplementary Information

Below is the link to the electronic supplementary material.

Supplementary Material 1

Supplementary Material 2

## Data Availability

The data that support the findings of this study are available on request from the corresponding author. The data are not publicly available due to privacy or ethical restrictions.
