# Supplementary material for: Maternal immunity and post-vaccination sero-monitoring in small ruminants for peste des petits ruminants eradication in North Shewa, Ethiopia
Source: Sci Rep. 2026 Feb 27;16:11275. doi: 10.1038/s41598-026-41977-3 (PMC13049061; doi:10.1038/s41598-026-41977-3)
Supplement: Supplementary file 1 — Supplementary Material 1 [file 41598_2026_41977_MOESM1_ESM.docx]

**Questionnaire for Assessing Health, Management, and Peste des Petits Ruminants (PPR) Risk Factors in Small Ruminants**

How are you? My name is ____________________, and I am part of a research team conducting a study on the health and management of small ruminants, specifically sheep and goats, in this area. The main objective of this study is to assess the impact of the risk-based vaccination strategy (RBVS) on the control and eradication of Peste des Petits Ruminants (PPR). To do this, we are collecting detailed information about individual animals and their flocks, including their origin (district, PA, and village), age, sex, species, body condition score, and vaccination history. This data will help us identify patterns of disease and improve animal health services and vaccination programs in your region. Please be assured that all the information you provide will be kept strictly confidential. Your personal details and responses will only be used for research purposes and will not be shared outside the research team. The data will be stored securely and accessed only by authorized personnel. Your participation is entirely voluntary, and you are free to decline to answer any question or withdraw from the interview at any time without any consequences. If you agree to respond to the following questions, please sign it in the space provided below. Please feel free to ask any questions you might have before we begin.

Name of the respondant_____________________________Signiture___________________

**Section 1: General Information**

**1.1 Enumerator Details**

- Enumerator Name: ___________________________
- Date of Data Collection: ___ / ___ / ________
- Questionnaire ID/Code: ______________________

**1.2 Location Details**

1. Zone Name: ___________________________
2. District (Woreda): ______________________
3. Peasant Association (PA): ________________
4. Village: _________________________

**Section 2: Flock Information**

**2.1 Flock Identification**

1. Village name: _________________________________
2. Owner/Household Head Name: ________________
3. Contact (if applicable): _____________________

**2.2 Flock Vaccination Status**

1. Has your flock ever been vaccinated against PPR?
   - Yes
   - No
2. If yes, when was the last vaccination conducted?
   - Date (approx.): ___ / ___ / ______

**Section 3: Animal-Level Information**

*(Fill this section per animal in the flock, use a table for easier data entry)*

| **Animal ID** | **Species (Sheep/Goat)** | **Age (Months)** | **Sex (M/F)** | **Body Condition Score (1–5)** | **Origin** | | |
| --- | --- | --- | --- | --- | --- | --- | --- |
|  |  |  |  |  | **District** | **Peasant Association** | **Village** |
|  |  |  |  |  |  |  |  |
|  |  |  |  |  |  |  |  |
|  |  |  |  |  |  |  |  |
|  |  |  |  |  |  |  |  |
|  |  |  |  |  |  |  |  |
|  |  |  |  |  |  |  |  |
|  |  |  |  |  |  |  |  |
|  |  |  |  |  |  |  |  |
|  |  |  |  |  |  |  |  |
|  |  |  |  |  |  |  |  |

**Section 4: Animal Acquisition History**

1. **Was any new animal introduced to this flock in the past 12 months?**
   - Yes
   - No
2. **If yes, what was the source of the new animal(s)?** *(Check all that apply)*
   - Gift
   - Breeding loan/share
   - Purchase
   - Others (specify): ______________________
3. **Number of animals introduced by source (if known):**

| **Source** | **Number Introduced** |
| --- | --- |
| - Gift |  |
| - Breeding |  |
| - Purchase |  |
| - Other |  |

1. **From where was the animal acquired? (Specify location if known)**
   - District: ___________________
   - PA: _________________________
   - Village: ____________________

**Section 5: Additional Flock Data**

1. Total number of small ruminants in the flock:
   - Sheep: _______
   - Goats: _______
2. Approximate age distribution (number of animals by age group):
   - Less than 6 months: ______
   - 6–12 months: ______
   - 1–2 years: ______
   - More than 2 years: ______
3. Does the flock typically mix with other flocks (e.g., communal grazing)?
   - Yes
   - No
   - If yes, specify how frequently and with how many other flocks: __________

**Section 6: Notes and Observations**

- Any notable observations about flock management, animal health, or environmental conditions:
- Respondent’s comments:
