# Supplementary material for: Maternal immunity and post-vaccination sero-monitoring in small ruminants for peste des petits ruminants eradication in North Shewa, Ethiopia
Source: Sci Rep. 2026 Feb 27;16:11275. doi: 10.1038/s41598-026-41977-3 (PMC13049061; doi:10.1038/s41598-026-41977-3)
Supplement: Supplementary file 2 — Supplementary Material 2 [file 41598_2026_41977_MOESM2_ESM.docx]

## **Small ruminant flock antibody immunity against PPRV**

## A total of 508 serum samples were collected from 22 villages across eight peasant associations in four districts. Of these, 332 tested positives for PPRV antibodies, resulting in an overall individual-level antibody immunity of 65.35% (95% CI: 61–70%), which is below the recommended flock immunity threshold of 80%, the level generally required to reduce infection transmission sufficiently to eliminate the virus. At the flock level, considering each village as a single flock, only 6 of the 22 flocks (22.27%) had ≥80% of animals with detectable antibodies. The highest flock-level antibody immunity was observed in Arkisekelo (92.74%, 95% CI: 74–99%), while the lowest was in Keyafer (22.73%, 95% CI: 8–45%). Overall, 72.73% of the flocks had less than 80% antibody coverage, highlighting areas where additional vaccination may be needed to enhance population-level immunity (Table S1).

## **Table S1:** Flock level antibody immunity against PPRV in vaccinated small ruminants

| **Districts** | **Peasant Associations** | **Villages** | **No. Sampled** | **No. Positive** | **Prevalence (%)** | **95%CI** | |
| --- | --- | --- | --- | --- | --- | --- | --- |
|  |  |  |  |  |  | **Lower** | **Upper** |
| Basonawerena | Abamote | Derek-Wenz | 25 | 20 | 80 | 0.59 | 0.93 |
|  |  | Jilobado | 26 | 22 | 84.62 | 0.65 | 0.96 |
|  |  | Kosso | 24 | 20 | 83.33 | 0.63 | 0.95 |
|  | Kormargefiya | Arkisekelo | 25 | 23 | 92.74 | 0.74 | 0.99 |
|  |  | Milkiy | 28 | 18 | 78.26 | 0.44 | 0.81 |
|  |  | Kormargefiya | 23 | 13 | 56.52 | 0.35 | 0.77 |
| Kewet | Tere | Tere 2 | 24 | 20 | 83.33 | 0.63 | 0.95 |
|  |  | Jaber | 20 | 15 | 75 | 0.51 | 0.91 |
|  |  | Tere 1 | 27 | 17 | 62.96 | 0.42 | 0.81 |
|  | Yelen | Guduamaba | 20 | 8 | 40 | 0.19 | 0.64 |
|  |  | Wacho | 19 | 12 | 63.16 | 0.38 | 0.84 |
| Shewa-robit | Wanza | Wanza | 25 | 13 | 52 | 0.31 | 0.72 |
|  |  | Ashewa | 27 | 21 | 77.77 | 0.58 | 0.91 |
|  | Wustimbay | Wustimboy | 22 | 15 | 68.18 | 0.45 | 0.86 |
|  |  | Juvamba | 23 | 7 | 30.43 | 0.13 | 0.53 |
| Menz-mama | Keyafer | Keyafer | 22 | 5 | 22.73 | 0.08 | 0.45 |
|  |  | Misreta | 22 | 13 | 59.09 | 0.36 | 0.79 |
|  |  | Zole | 23 | 16 | 69.57 | 0.47 | 0.87 |
|  | Zeram | Godere | 24 | 14 | 58.33 | 0.37 | 0.78 |
|  |  | Kesochbado | 22 | 11 | 50 | 0.28 | 0.72 |
|  |  | Ankelafign | 22 | 11 | 50 | 0.28 | 0.72 |
|  |  | Tachgodere | 20 | 18 | 90 | 0.68 | 0.99 |
| Total | |  | 508 | 332 | 65.35 | 0.61 | 0.7 |
